# Prevention of Oxygen-Induced Inflammatory Lung Injury by Caffeine in Neonatal Rats

**DOI:** 10.1155/2020/3840124

**Published:** 2020-08-07

**Authors:** Stefanie Endesfelder, Evelyn Strauß, Ivo Bendix, Thomas Schmitz, Christoph Bührer

**Affiliations:** ^1^Department of Neonatology, Charité-Universitätsmedizin Berlin, Berlin, Germany; ^2^Department of Pediatrics I, Neonatology and Experimental Perinatal Neurosciences, University Hospital Essen, University Duisburg-Essen, Essen, Germany

## Abstract

**Background:**

Preterm birth implies an array of respiratory diseases including apnea of prematurity and bronchopulmonary dysplasia (BPD). Caffeine has been introduced to treat apneas but also appears to reduce rates of BPD. Oxygen is essential when treating preterm infants with respiratory problems but high oxygen exposure aggravates BPD. This experimental study is aimed at investigating the action of caffeine on inflammatory response and cell death in pulmonary tissue in a hyperoxia-based model of BPD in the newborn rat. *Material/Methods*. Lung injury was induced by hyperoxic exposure with 80% oxygen for three (P3) or five (P5) postnatal days with or without recovery in ambient air until postnatal day 15 (P15). Newborn Wistar rats were treated with PBS or caffeine (10 mg/kg) every two days beginning at the day of birth. The effects of caffeine on hyperoxic-induced pulmonary inflammatory response were examined at P3 and P5 immediately after oxygen exposure or after recovery in ambient air (P15) by immunohistological staining and analysis of lung homogenates by ELISA and qPCR.

**Results:**

Treatment with caffeine significantly attenuated changes in hyperoxia-induced cell death and apoptosis-associated factors. There was a significant decrease in proinflammatory mediators and redox-sensitive transcription factor NF*κ*B in the hyperoxia-exposed lung tissue of the caffeine-treated group compared to the nontreated group. Moreover, treatment with caffeine under hyperoxia modulated the transcription of the adenosine receptor (Adora)1. Caffeine induced pulmonary chemokine and cytokine transcription followed by immune cell infiltration of alveolar macrophages as well as increased adenosine receptor (Adora1, 2a, and 2b) expression.

**Conclusions:**

The present study investigating the impact of caffeine on the inflammatory response, pulmonary cell degeneration and modulation of adenosine receptor expression, provides further evidence that caffeine acts as an antioxidative and anti-inflammatory drug for experimental oxygen-mediated lung injury. Experimental studies may broaden the understanding of therapeutic use of caffeine in modulating detrimental mechanisms involved in BPD development.

## 1. Introduction

Through intensive medical care, the survival rate of extremely and very small premature infants has improved, whereas the incidence of developmental deficits and the risk of chronic diseases is almost unchanged [1]. Prematurely born children are already exposed to higher oxidative stress at birth [2], and due to the fundamental immaturity of the organs and organ systems, as well as their immature antioxidant defense [3], they are also unable to adapt to it adequately. An additive oxygen therapy for the treatment of respiratory instabilities can increase this effect [4]. Currently, long-term observations of premature infants are increasingly indicating that chronically increased oxidative stress levels could be the cause of chronic diseases in adulthood, e.g., metabolic syndrome, diabetes, and respiratory diseases in consequence of bronchopulmonary dysplasia (BPD) [5–7]. Oxidative stress in correlation with the immature developmental stage of the lungs and the influence of exogenous factors, such as mechanical stress and post- or antenatal infections, are among the most important reasons for the complex and multifactorial mechanisms of BPD [8–10]. BPD is associated with a higher risk of short- and long-term respiratory diseases and morbidity, accompanied by impaired lung structure and function. To date, no effective or preventive therapy of BPD is available.

Well-tolerated therapeutic strategies exist to alleviate premature lung injury such as corticosteroids [11, 12], surfactant [13], noninvasive respiratory assistance and lung protective ventilation procedures [14], careful oxygen usage [15], and antioxidants [16]. Most therapeutic approaches exhibit no effects in the symptomatic treatment or prevention of BPD, with the exception of the antioxidants vitamin A and caffeine, although efficacy is not fully established. A small but beneficial effect of vitamin A in lowering rates of BPD has been found in preterm neonates born before 29 weeks' gestation [17, 18]. Data from initial clinical studies of caffeine therapy show a reduction in BPD rates [19, 20] and significant improvement of lung function [21]. The discussed early prophylactic caffeine treatment before extubation is partly associated with a reduced incidence of BPD [22–24] but also linked with a higher mortality compared to a later start of caffeine administration (>3 postnatal days) [25, 26].

The molecular mechanisms of BPD are complex. Baro- and volutrauma and oxygen toxicity are thought to be the main triggers [8]. Morphological changes due to oxidative stress result in the disruption of lung development [27], accompanied by increased proinflammatory cytokine and chemokine expression and immune cell infiltration in lung tissue [28–30]. Apoptosis and proliferation play an important role in normal cell turnover and tissue development. Mechanical ventilation and elevated oxygen concentration increase the numbers of apoptotic cells in human foetal lungs [31, 32].

Hyperoxia-based BPD models [8, 33] are essential for better understanding and treating long-term pulmonary sequelae, and similar histological changes occur in hyperoxia-exposed rodent pups and human preterm infants [34–36]. Newborn postnatal rats correlate in terms of developmental stages at birth with the saccular phase (E18-P4; human 24-38 weeks of gestation age) and then with the first/classical phase of alveolarization (P4-P21; human 36 weeks of gestation age to postnatal age), which overlaps with the second/continuous phase of alveolarization (P14-P60) [37].

Oxygen toxicity is mediated by the production and accumulation of excessive reactive oxygen species (ROS), causes oxidative stress [38], and depleted antioxidant levels [39]. Supraphysiologic oxygen can cause oxygen toxicity and inflammation, ultimately leading to hyperoxic lung injury and impairment of lung development caused of an influx of inflammatory cells, increased pulmonary permeability, simplification of lung structure, and cell death [9, 40]. Damage to the developing lung may be caused directly by the generation of ROS as cell toxin or indirectly through the activation of inflammatory responses [41, 42]. The inflammatory reaction is triggered by hyperoxia in turn worsens the oxidative toxicity [43]. It has not yet been conclusively determined whether under oxidative stress caffeine acts as an antagonist to adenosine receptors [44] or through antioxidant properties [39], anti-inflammatory effects [45, 46], and/or the reduction of endoplasmic reticulum stress [47].

Therefore, this study is aimed at investigating the action of caffeine on main mediators of inflammatory response and cell death in pulmonary tissue in a hyperoxia-based model of BPD in the newborn rat.

## 2. Materials and Methods

### 2.1. Ethics Statement

Time-pregnant *Wistar* rat dams were obtained from the Department of Experimental Medicine (FEM, Charité-Universitätsmedizin Berlin, Germany). The adult rats were housed in individual cages under environment-controlled conditions with a constant 12 h/12 h light/dark cycle, ambient temperature, and relative humidity of 40-60% with *ad libitum* access to the same food and water. All animal experimental procedures were approved by the local animal welfare authorities (LAGeSo, approval number G-0088/16) and followed institutional guidelines as well as ARRIVE guidelines.

### 2.2. Oxygen Exposure and Drug Administration

As previously described [39], pups from different litters and both sexes were pooled and randomized within 12 h of birth and returned to the dams. Sample size calculation was performed in G∗Power V3.1.2 [48]. The newborn rats were randomly assigned to room air (normoxia, NO) or oxygen-enriched atmosphere (hyperoxia, HY) treatment. The pups in hyperoxia subgroups were reared with the dams in an atmosphere containing 80% oxygen (OxyCycler BioSpherix, Lacona, NY) from postnatal day (P)0 to P3 (*n* = 7-8) or P0 to P5 (*n* = 6-8); in parallel, the pups in normoxia groups were reared with the dams under room air conditions. To avoid oxygen toxicity in the nursing mothers, they were rotated between the hyperoxic and normoxic litters every 24 h. The rats were divided into four groups, each for the same exposure times to (i) normoxia (NO, control group): 21% oxygen application of vehicle (phosphate-buffered saline (PBS)); (ii) normoxia with caffeine (NOC): 21% oxygen with caffeine (10 mg/kg, Sigma, Steinheim, Germany); (iii) hyperoxia (HY): 80% oxygen with vehicle (PBS); and (iv) hyperoxia with caffeine (HYC): 80% oxygen with caffeine (10 mg/kg). 10 mg/kg of pure caffeine is equivalent to 20 mg/kg caffeine citrate, which is used clinically. Rat pups received either drug or vehicle injection intraperitoneally (i.p.) as a fixed proportion of their body weight (100 *μ*l/10 g) every 48 h beginning on the day of birth (P0). Caffeine or vehicle was administered to the pups with a total of three postnatal days of oxygen exposure (P0-P3) on the day of birth (P0) and on P2 and for the rat pups with a total of five days of postnatal oxygen exposure (P0-P5) on the day of birth (P0) and on P2 and P4. The rat pups were examined after the oxygen exposure (P3, P5) either directly or after recovery in room air at P15 (P3_P15, P5_P15). No pups died during hyperoxia. Caffeine plasma concentrations and weight profiles were determined and presented in the previous work [39].

### 2.3. Tissue Preparation

At the experimental endpoints (P3; P3_P15; P5; P5_P15), rat pups were anaesthetized with an *i.p.* injection of ketamine (100 mg/kg), xylazine (20 mg/kg), and acepromazine (3 mg/kg) and then transcardially perfused, as previously described [39]. The heart lung block was immediately removed, and the lungs were snap-frozen in liquid nitrogen and stored at -80°C. The perfusion was carried out with PBS (pH 7.4) for the molecular analysis and for immunohistochemical analysis followed by perfusion with 4% paraformaldehyde (pH 7.4); the lungs were postfixed at 4°C for 1 day, embedded in paraffin, and processed for histological staining.

### 2.4. RNA Extraction and Quantitative Real-Time PCR

Pulmonary tissue procurement has already been described [39]. Briefly, total RNA was isolated from snap-frozen tissue by acidic phenol/chloroform extraction (peqGOLD RNAPure™; PEQLAB Biotechnologie, Erlangen, Germany), and 2 *μ*g of RNA was DNase treated and reverse-transcribed. The PCR products of adenosine A1 receptor (Adora1), adenosine A2a receptor (Adora2a), adenosine A2b receptor (Adora2b), apoptosis inducing factor, mitochondria associated (AIF), caspase 3 (Casp3), CD74 molecule (CD74), C-X-C motif chemokine ligand 1 (CINC-1), glutamate-cysteine ligase catalytic subunit (GCLC), hypoxanthine phosphoribosyltransferase 1 (HPRT), interferon gamma (IFN*γ*), interleukin 1 alpha (IL-1*α*), interleukin 1 beta (IL-1*β*), interleukin 10 (IL-10), C-C motif chemokine ligand 2 (MCP-1), Macrophage migration inhibitory factor (MIF), C-X-C motif chemokine ligand 2 (MIP-2), nuclear factor kappa B subunit 1 (NF*κ*B1), nuclear factor kappa B subunit 2 (NF*κ*B2), and tumor necrosis factor (TNF*α*) were quantified in real time with the sequences summarized in Table 1. PCR and detection were performed with qPCR BIO Mix Hi-ROX (NIPPON Genetics Europe, Düren, Germany) with HPRT used as an internal reference. The expression of target genes was analyzed with the StepOnePlus real-time PCR system (Applied Biosystems, Carlsbad, CA, USA) according to the 2^-*ΔΔ*CT^ method [49].

### 2.5. Protein Extraction

The snap-frozen lung tissue was chopped in a radioimmunoprecipitation assay buffer (RIPA; Thermo Fisher Scientific, Waltham, MA, USA), centrifuged at 3000× *g* for 10 min at 4°C, and the supernatant of homogenized lung tissue was obtained. Protein concentrations were determined using the Pierce BCA kit (Pierce/Thermo Fisher Scientific, Rockford, IL, USA) as described in [50].

### 2.6. Enzyme-Linked Immunosorbent Assay (ELISA)

Tumor necrosis factor *α* (TNF*α*) concentration was analyzed in samples of lung homogenate using rat TNF*α*/TNFSF1A Quantikine ELISA Kit (R&D Systems GmbH, Wiesbaden-Nordenstadt, Germany) according to the manufacturer's instructions and as described in [51]. TNF*α* concentration was estimated from the standard curve and expressed as picogram per milligram protein.

### 2.7. Immunohistochemistry

Paraffin-embedded lung sections were deparaffinized in Roti-Histol (Carl Roth, Karlsruhe, Germany), rehydrated in ethanol, and subjected to immunostaining. Antigen retrieval was done in a heated citrate buffer at pH 6.0 for 20 min. To block unspecific proteins, slices were incubated in PBS 2% goat serum, 1% BSA, 0.1% Triton X-100, 0.05% Tween 20, and 0.1% bovine gelatin. Primary antibodies were diluted in antibody diluent (Zymed Laboratories, San Francisco, CA) and incubated with the sections overnight at 4°C. Sections were stained for anti-DNA fragmentation factor subunit beta (DFFB, 5 *μ*m section, 1 : 250, Bioss, Woburn, MA, USA), for anti-myeloperoxidase (MPO, 10 *μ*m section, 1 : 200, Thermo Fisher Scientific), and for ED1 (CD68, 10 *μ*m section, 1 : 200, Abcam, Cambridge, MA, USA). For detection, secondary Alexa Fluor 488-conjugated goat anti-mouse IgG (Thermo Fisher Scientific) or Alexa Fluor 488-conjugated goat anti-rabbit IgG (Thermo Fisher Scientific) was applied at a dilution of 1 : 200 in antibody diluent (Zymed Laboratories) and incubated at room temperature for 1 h. Slides were counterstained with 4,6-diamidino-2-phenylindole (DAPI, 1 : 2000 in PBS, Sigma) for 10 min and mounted with mounting media (Shandon Immu-Mount, Thermo Fisher Scientific). Sections were analyzed blind using a Keyence compact fluorescent microscope BZ 9000 with BZ-II Viewer software and BZ-II Analyzer software (Keyence, Osaka, Japan). Green fluorescent-positive cells were counted in four (DFFB, 200x magnification) or six (MPO and ED1, 100x magnification) nonoverlapping separate fields per animal, and mean values of all images were used for statistical analysis.

### 2.8. Statistical Analyses

Box and Whisker plots represent the interquartile range (box) with the line representing the median, whereas whiskers show the data variability outside the upper and lower quartiles. Groups were compared using one-way analysis of variance (ANOVA) based on a partially non-Gaussian distribution with the Kruskal-Wallis test. Multiple comparisons of means were carried out using Dunn's *post hoc* test. A *p* value of <0.05 was considered significant. All graphics and statistical analyses were performed using the GraphPad Prism 8.0 software (GraphPad Software, La Jolla, CA, USA).

## 3. Results

### 3.1. Caffeine Inhibits Cell Apoptosis in Pulmonary Tissue Induced by Hyperoxia

**Figure 1:**
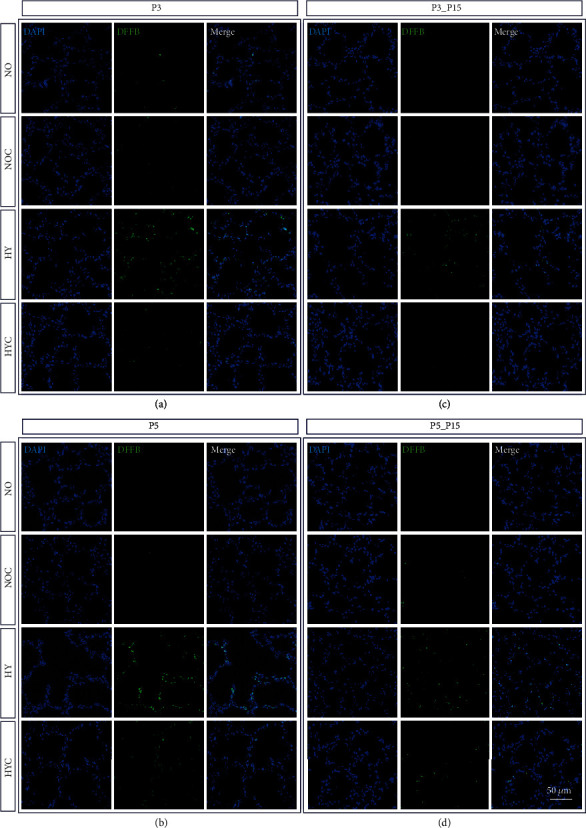
Representative photomicrographs of immunohistochemical staining of DFFB in the lungs of rat pups exposed to normoxia (NO) or hyperoxia (HY) compared to rat pups treated with caffeine (NOC, HYC). Examinations were performed at postnatal day 3 (P3 (a)) and P5 (b), or after recovery after 3-day exposure at P15 (c) or after 5-day exposure at P15 (d). Immunofluorescent images indicated DFFB (green) and nuclei (blue, DAPI). Scale bars represent 50 *μ*m.

**Figure 2:**
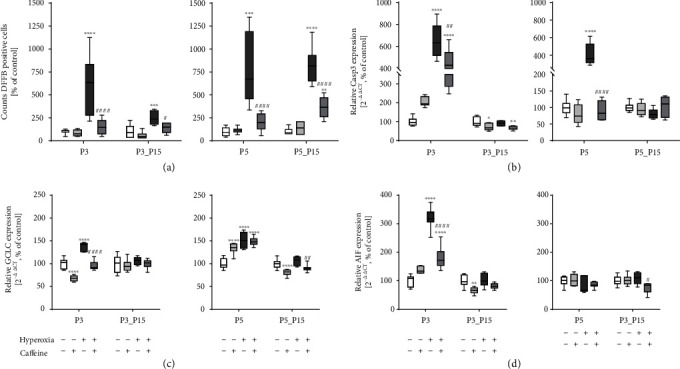
Quantitative analysis of (a) DFFB+ cell counts showed a marked increase in lung tissue samples of rat pups after acute oxygen exposure at P3 and P5 (deep dark grey bars) whereas caffeine treatment reduced apoptotic cells (dark grey bars). Cell death rate remained elevated even after recovery (P15). Caffeine treatment under room air (light grey bars) demonstrated no modulation of cell death. Hyperoxia within the first days of life accompanied by an increased cell death rate led to enhanced gene expression of (b) Casp3, (c) GCLC, and (d) AIF. Caffeine counteracted this. Quantification of lung homogenates was performed with qPCR for 3 days' postnatal oxygen exposure (P3) and recovery (P3_P15) and 5 days' postnatal oxygen exposure (P5) and recovery (P5_P15), respectively. Data are normalized to the level of rat pups exposed to normoxia at each time point (control 100%, white bars), and the 100% values are 4.3 (P3), 1.7 (P3_P15), 3.1 (P5), and 0.7 (P5_P15) cells per mm^2^, respectively. *n* = 6-8/group. ^∗^*p* < 0.05, ^∗∗^*p* < 0.01, ^∗∗∗^*p* < 0.001, and ^∗∗∗^*p* < 0.0001 vs. control; ^#^*p* < 0.05, ^##^*p* < 0.01, and ^####^*p* < 0.0001 vs. hyperoxia (ANOVA, Kruskal-Wallis, Dunn's *post hoc* test).

The effects of caffeine on the histopathological findings of rat pups with hyperoxia-induced lung tissue injury are shown in Figure 1. To gain insight into the effect of caffeine on cell survival during hyperoxia-induced lung injury, apoptosis in the lung tissue was evaluated by DFFB staining. Cell apoptosis in the lung tissue was induced by hyperoxia and was detected immediately after the termination of oxygen exposure after 3 and 5 days and remained consistent even after recovery in ambient air until postnatal day 15 compared to animals exposed to room air. At all times of examination, there was a significant decrease of apoptotic cells in the caffeine-treated hyperoxia group compared to the vehicle-treated hyperoxia group (Figures 1 and 2(a)). In accordance with the IHC results in neonatal rat lungs, the mRNA content of the effector caspase Casp3 and caspase-independent AIF increased in direct response to hyperoxia (P3 and/or P5; Figure 2(b) and (d)). The level of mRNA encoding GCLC, a rate-limiting enzyme subunit of glutathione synthesis, was upregulated in a similar way (Figure 2(c)). Hyperoxia-induced transcription of cell death-associated mediators was significantly weakened by caffeine. Caffeine under normoxia exposure had no effect of mRNA expression of the cell death associated mediators Casp3, AIF, and GCLC, compared to the control group (Figure 2(b) and (d)).

### 3.2. Hyperoxia-Induced Immune Cell Infiltration Is Inhibited by Caffeine

**Figure 3:**
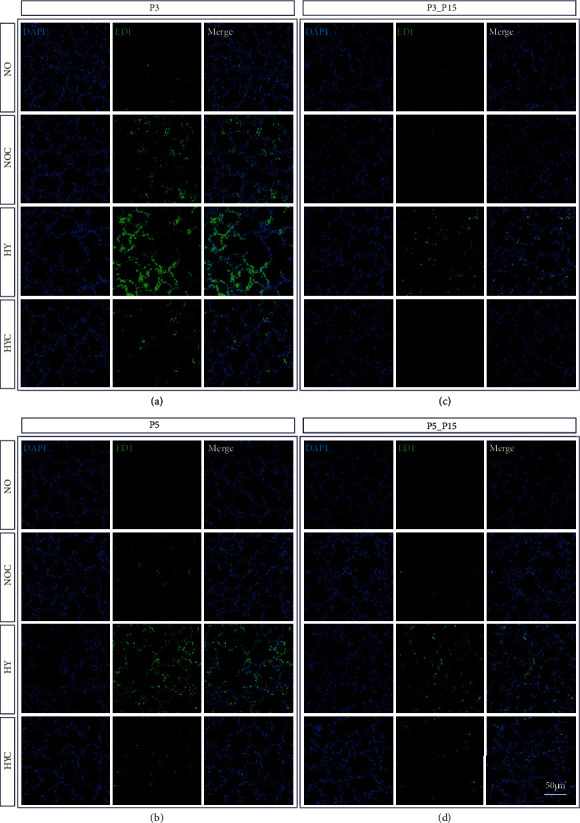
Representative micrographs of immunohistochemical staining of ED1 (CD68) in the lungs of rat pups exposed to normoxia (NO) or hyperoxia (HY) compared to rat pups treated with caffeine (NOC, HYC). Examinations were performed at postnatal day 3 (P3 (a)) and P5 (b), or after recovery after 3-day exposure at P15 (c) or after 5-day exposure at P15 (d). Immunofluorescent images indicated ED1 (green) and nuclei (blue, DAPI). Scale bars represent 100 *μ*m.

**Figure 4:**
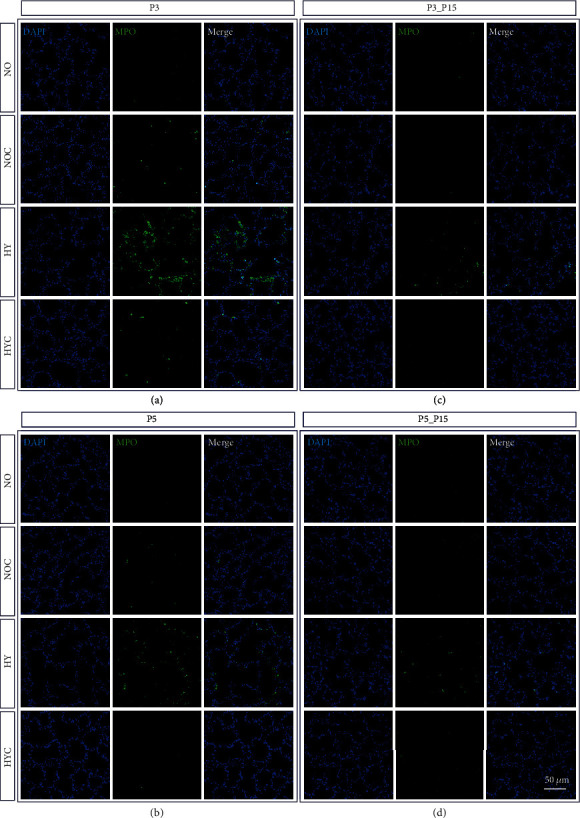
Representative micrographs of immunohistochemical staining of MPO in the lungs of rat pups exposed to normoxia (NO) or hyperoxia (HY) compared to rat pups treated with caffeine (NOC, HYC). Examinations were performed at postnatal day 3 (P3 (a)) and P5 (b), or after recovery after 3-day exposure at P15 (c) or after 5-day exposure at P15 (d). Immunofluorescent images indicated MPO (green) and nuclei (blue, DAPI). Scale bars represent 100 *μ*m.

**Figure 5:**
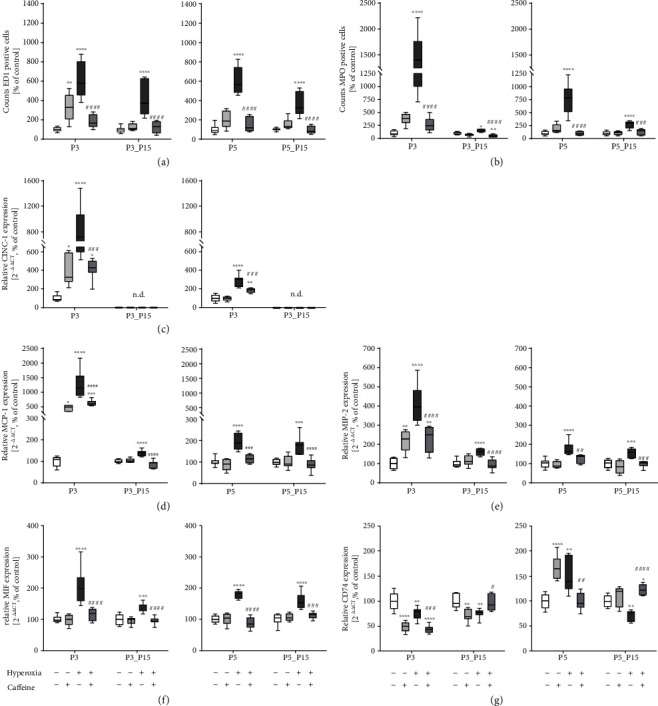
Quantitative analysis of (a) ED1+ and (b) MPO+ cell counts showed a marked accumulation in lung tissue samples of rat pups after acute oxygen exposure at P3 and P5 (deep dark grey bars) whereas caffeine treatment inhibited (a) macrophage (ED1) and (b) neutrophil (MPO) infiltration (dark grey bars). Macrophage infiltration remained elevated even after recovery (P15). Caffeine treatment under room air (light grey bars) increased counts of macrophages at P3. Hyperoxia exposure of newborn rat pups, characterized by an overwhelming immune cell influx, led to increased chemokine gene expression of (c) CINC-1, (d) MCP-1, and (e) MIP-2. Chemokine transcription is inhibited by caffeine. Caffeine under normoxic exposure increased chemokine transcription at P3. CINC-1 is not detectable (n.d.) at P15. Caffeine alleviated the hyperoxia-induced gene expression of (f) MIF, whereas hyperoxia modulated expression of the MIF receptor (g) CD74, and caffeine counteracted this. Quantification of lung homogenates was performed with qPCR for 3 days' postnatal oxygen exposure (P3) and recovery (P3_P15) and 5 days' postnatal oxygen exposure (P5) and recovery (P5_P15), respectively. Data are normalized to the level of rat pups exposed to normoxia at each time point (control 100%, white bars), and the 100% values for ED1/MPO are 22.4/12.7 (P3), 7.6/12.5 (P3_P15), 15.2/12.0 (P5), and 7.1/10.9 (P5_P15) cells per mm^−2^, respectively. *n* = 6-8/group. ^∗^*p* < 0.05, ^∗∗^*p* < 0.01, ^∗∗∗^*p* < 0.001, and ^∗∗∗∗^*p* < 0.0001 vs. control; ^#^*p* < 0.05, ^##^*p* < 0.01, ^###^*p* < 0.001, and ^####^*p* < 0.0001 vs. hyperoxia (ANOVA, Kruskal-Wallis, Dunn's *post hoc* test).

Immunohistochemistry was used to assess the infiltration of the lungs by inflammatory cells. The 3-day and 5-day hyperoxic exposure exhibited significant inflammatory cell infiltration, as compared to controls (Figures 3 and 4), and a quantitative increase of ED1-positive alveolar macrophages and MPO-positive neutrophils (Figure 5(a) and (b)). Alveolar macrophages and neutrophils in hyperoxic lungs were still increased after recovery (Figure 5(a)). Following caffeine intervention, the number of infiltrating immune cells (i.e., ED1+ and MPO+ cells) was significantly decreased (Figures 3–5(a), (b)). Caffeine under normoxic conditions produced a significant increase in alveolar macrophages on postnatal day three (Figure 5(a)).

Chemokines play an important role in mediating immune cell recruitment in the lung during hyperoxic exposure [52]. As shown in Figure 5(c)–(e), following hyperoxia, we observed a robust increase in the expression of the cytokine-induced chemokine CINC-1, of the monocytes chemotactic chemokine MCP-1, and of the neutrophil chemotactic chemokine MIP-2 which were largely increased in the hyperoxia group compared to the normoxic group, which was ameliorated by caffeine treatment (Figure 5(c)–(e)). Notably, the increase was far more pronounced at the immature age P3 compared to the later postnatal age P5. Surprisingly, caffeine in room air-exposed rat lungs significantly increased chemokine mRNA expression at P3 (Figure 5(c)–(e)). Following hyperoxia, we detected an increase of the inflammatory mediator MIF at any time point (Figure 5(f)). Transcript levels encoding for CD74 as part of MIF receptor complex were decreased under hyperoxic exposure on postnatal day 3 (P3), and expression remained decreased even after recovery in ambient air (P3_P15, Figure 5(g)). Interestingly, CD74 mRNA was significantly elevated after five days of hyperoxia (P5) and decreased until postnatal day 15 (P5_P15; Figure 5(g)) compared to lung tissue from normoxic rat pups. Caffeine inhibited MIF transcription, which was upregulated by hyperoxia, at any time point (Figure 5(f)). In the hyperoxia-exposed lungs, caffeine decreased CD74 transcription at P3 and P5, but after recovery, it caused an increase of CD74 expression at P15 (P3/P5_P15; Figure 5(g)).

### 3.3. Caffeine Reduces Inflammatory Response Induced by Hyperoxia In Vivo

**Figure 6:**
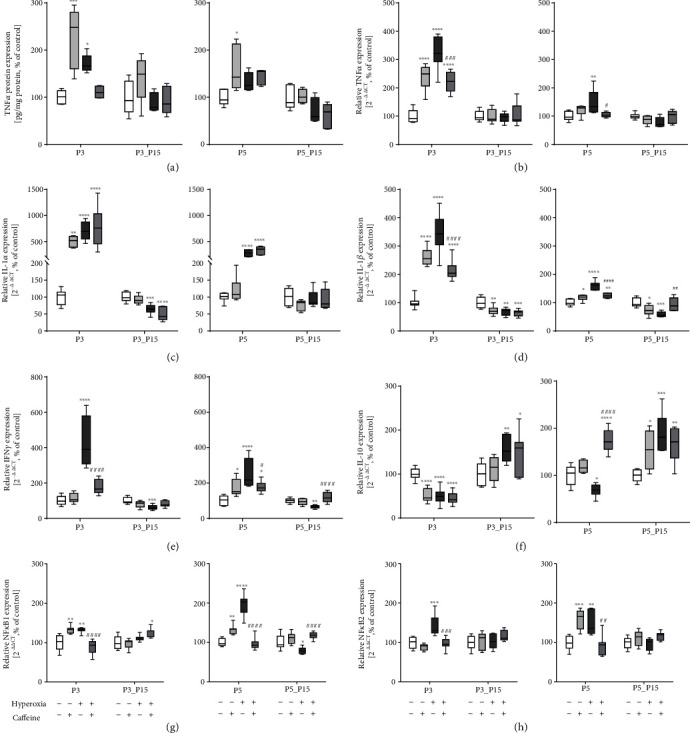
Hyperoxia exposure of newborn rat pups is characterized by modulation of cytokine response and regulation of redox-sensitive transcription factors. High oxygen led to increased proinflammatory cytokine expression of (a) TNF*α* (protein), (b) TNF*α* (RNA), (c) IL-1*α*, (d) IL-1*β*, and (e) IFN*γ*. Anti-inflammatory cytokine expression of (f) IL-10 is suppressed and redox-sensitive transcription factors (g) NF*κ*B1 and (h) NF*κ*B2 are increased. Caffeine counteracted this. Caffeine under normoxic exposure demonstrated a massive modulation of proinflammatory cytokine expression. Quantification of lung homogenates by qPCR for 3 days' postnatal oxygen exposure (P3) and recovery (P3_P15) and 5 days' postnatal oxygen exposure (P5) and recovery (P5_P15), respectively. Data are normalized to the level of rat pups exposed to normoxia at each time point (control 100%, white bars). The 100% values of TNF*α* protein are 4.4 (P3), 5.6 (P3_P15), 3.5 (P5), and 7.2 (P5_P15) pg per ml, respectively. *n* = 7-8/group (qPCR); *n* = 5/group (ELISA). ^∗^*p* < 0.05, ^∗∗^*p* < 0.01, ^∗∗∗^*p* < 0.001, and ^∗∗∗∗^*p* < 0.0001 vs. control; ^#^*p* < 0.05, ^##^*p* < 0.01, and ^####^*p* < 0.0001 vs. hyperoxia (ANOVA, Kruskal-Wallis, Dunn's *post hoc* testq).

Given that caffeine treatment reduced cell apoptosis and immune cell infiltration in the developing lung, next, we analyzed the expression of proinflammatory cytokines in order to gain insight into the potential anti-inflammatory action of caffeine. In response to hyperoxia, the concentration of TNF*α* significantly increased in lung tissue determined by ELISA at P3 only (Figure 6(a)) and was confirmed for the mRNA expression at P3 and P5 (Figure 6(b)). For both protein and mRNA, we observed an induction of TNF*α* by caffeine without additional oxygen (P3 and/or P5; Figure 6(a) and (b)). In addition, hyperoxia-exposed rat pups had increased IL-1*α*, IL-1*β*, and IFN*γ* mRNA in lung homogenates compared to control rats at P3 and P5 (Figure 6(c)–(e)). In hyperoxic lung-tissue, proinflammatory cytokines were no longer detected after the recovery phase (P15), but rather an exhaustion of mRNA transcription was detected (IL-1*α*, IL-1*β*, and IFN*γ* at P3_P15 and/or P5_P15; Figure 6(a)–(e)). Again, in normoxic animals, caffeine induced proinflammatory cytokine responses for IL-1*α* (P3), IL-1*β* (P3 and P5), and IFN*γ* (P5) mostly at the acute time points (Figure 6(c)–(e)). There was a significant decrease of TNF*α*, IL-1*α*, IL-1*β*, and IFN*γ* in the lung tissues of hyperoxic caffeine-treated rat pups (Figure 6(a)–(e)).

The expression level of IL-10 as an anti-inflammatory cytokine was significantly lower in hyperoxia-exposed rats at P3 and P5, but was significantly increased after the recovery phase. While caffeine application reduced the expression at P3, an increase of IL-10 expression was observed at P5 (Figure 6(f)).

The transcription factor *NFκB* regulates the cellular response to oxidative and inflammatory stress [53]. Hyperoxia resulted in an increased mRNA expression of the subunits *NFκB1* and *NFκB2* (Figure 6(g) and (h)) acutely after oxygen exposure (P3 and P5), whereas caffeine treatment counteracted the hyperoxia-induced expression (Figure 6(g) and (h)). Here, too, we noted an induced effect for the transcription factor under ambient air for caffeine.

### 3.4. Expression Pattern of Adenosine Receptor Subtypes

**Figure 7:**
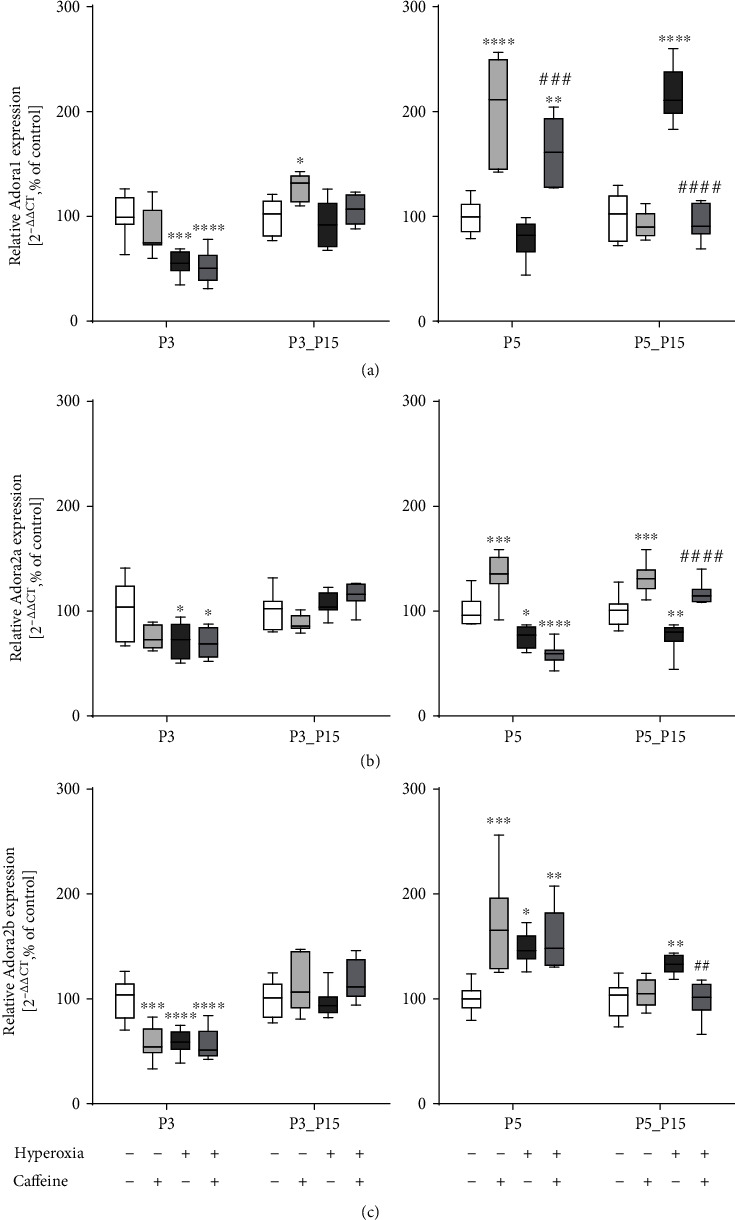
Hyperoxia and caffeine modulated gene expression of adenosine receptor subtypes (a) Adora1, (b) Adora2a, and (c) Adora2b. Quantification of lung homogenates was performed with qPCR for 3 days' postnatal oxygen exposure (P3) and recovery (P3_P15) and 5 days' postnatal oxygen exposure (P5) and recovery (P5_P15), respectively. Data are normalized to the level of rat pups exposed to normoxia at each time point (control 100%, white bars). *n* = 7-8/group. ^∗^*p* < 0.05, ^∗∗^*p* < 0.01, ^∗∗∗^*p* < 0.001, and ^∗∗∗∗^*p* < 0.0001 vs. control; ^##^*p* < 0.01, ^###^*p* < 0.001, and ^####^*p* < 0.0001 vs. hyperoxia (ANOVA, Kruskal-Wallis, Dunn's *post hoc* test).

The regulation of the adenosine receptor subtypes has been implicated in immune modulatory responses [54].  Figure 7 shows the effects of caffeine on the mRNA expression of adenosine receptor subtypes Adora1, Adora2a, and Adora2b, revealed by qPCR. Compared to control rat pups, there was a significant decrease in the expression levels of all investigated subtypes in lung tissue of hyperoxic animals at P3 (Figure 7(a)–(c)) and of Adora2a at P5 (Figure 7(b)), whereas Adora2b expression was increased at P5 (Figure 7(c)). After recovery under normoxic conditions, high oxygen exposure for five postnatal days increased Adora1 and Adora2b expression (Figure 7(a) and (c)), while the expression of Adora2a decreased (Figure 7(b)). Caffeine under control conditions induced the expression of all investigated receptor subtypes on postnatal day 5, and this effect continued for Adora2a until P15 (Figure 7(a)–(c)).

## 4. Discussion

In the present study, we demonstrated that treatment with caffeine represses hyperoxia-induced inflammation response and apoptosis in the developing lung, coinciding with a modulation of adenosine receptor subtypes, indicating that caffeine might act as a protective drug for hyperoxia-induced developmental lung injury.

One of the major effects of high oxygen is mainly characterized by excessive activation of pulmonary inflammatory response and cell death, which are regulated at several levels; production and accumulation of excessive ROS and the inflammatory reaction triggered by hyperoxia in turn worsen the oxidative toxicity [43]. This leads to diffuse alveolar damage, death of endothelial and epithelial cell, and immune cell influx into alveolar spaces [9, 55]. These bimodal processes of hyperoxia-induced lung damage are complex and are orchestrated by various overlapping or feedback pathways.

We demonstrate previously that caffeine diminishes oxidative stress and lung injury induced by hyperoxia [39], but little was known about effects on the secondary inflammatory response. Besides others [56, 57], this study confirmed that exposure to high oxygen led to a massive increase in cell death as an important pathological feature. Importantly, an increased cell death rate persists even after recovery in ambient air. Under prolonged hyperoxia until P3 and P5, an acute increase in cell death seems consistent, whereas after a recovery phase one would instead expect a normalization. The persistent cell death underlines DNA damage by oxidative stress [39] in connection with a sustained increase of alveolar macrophages and activated neutrophils at P15. Hyperoxia-induced lung injury is also the result of products of activated and recruited inflammatory cells into the lung tissue. The influx of neutrophils and monocytes into the lung is mediated by the degree of chemotactic activity such as induction of MCP-1, MIP-2, and MIF, which is highly elevated in hyperoxic lungs [52]. Proinflammatory cytokines, e.g., CINC-1 and the rat homologue of IL-8, are important as well in determining the levels of chemotactic activity. The reduction of oxidative stress and inhibition of immune cell infiltration with correlating downregulation of chemokines by caffeine were found to be consistent with the reduced rate of cell death. Furthermore, the determination of apoptosis-associated mediators also showed different expression levels most probably due to elevated oxidative stress. Effector caspase 3, a key enzyme in the execution of apoptosis, the caspase-independent AIF, and the catalytic subunit of glutamate cysteine ligase (GCLC) were clearly induced at the acute time points of P3 and P5. Initiation of apoptotic cell death is depending on oxygen concentration and duration of exposure [58, 59]. Since the dysregulation of cell death was abolished by treatment with caffeine, it may serve for pharmacological modification of long-term consequences of preterm birth on lung development and tissue repair.

Proinflammatory cytokines are important mediators in hyperoxia-induced inflammatory response, which exacerbate oxidative stress injury in the lung tissue. Hyperoxia *per se* provokes cytokine and chemokine release as part of the inflammatory response and increased pulmonary capillary permeability, triggering the infiltration of immune cells and inhibiting alveolarization [9, 60, 61]. Tracheal aspirates from preterm neonates with BPD higher concentrations of cytokines have been detected [62, 63] as well as increased neutrophil filtration, which remains detectable in the airways during the first weeks of life [64]. The inflammatory response in hyperoxia-induced lung injury models of rodents is comparable to that of children affected by BPD [45, 61, 65]. As a result of oxidative damage caused by hyperoxia [39], here, we observed a drastic infiltration of immune cells as well as an overexpression of proinflammatory cytokines and chemokines in the lung tissue directly after hyperoxic exposure (P3 and P5) and following normoxic recovery (P15). These pathological alterations were largely reversed by caffeine delivery. In most cases, the infiltrated cells are macrophages and neutrophils [45, 66]. The immune cell recruitment to inflammatory sites is regulated by chemokines [58]. Chemokine expression increased dramatically after the hyperoxic insult. After recovery in room air, the number of infiltrated cells decreased in line with the chemokines but did not reach the control level. Both aspects were consistent with the increased numbers of apoptotic cells with continued elevated levels of chemoattractants, but without further increased cytokine levels. Inflammatory factors, secreted by macrophages, destroy the microvascular integrity, increase the permeability, and induce aberrant angiogenesis followed by impaired alveolarization [67]. Adverse postnatal exposure to inflammation caused sustained immune regulation followed by prolonged inflammation and lung remodeling. Oxygen exposure of mouse pups during the first four days of life within the saccular phase is sufficient to achieve impaired lung maturation by simplifying the lung structure [68]. This change in pulmonary morphology has also been demonstrated in our previous study with newborn rats, with oxidative stress-damaged lung structure beyond the onset of alveolarization [69]. Accordingly, premature infants with BPD exhibited anatomical and functional lung deficits in adolescence and adulthood [70]. In our oxygen damage model, we compared a three-day oxygen exposure (P0 to P3) associated with the saccular developmental stage (E18 to P4) to the five-day exposure (P0 to P5), up to early alveolarization (initiating P4) of the lung. The resulting immune cell infiltration as well as the inflammatory response was more strengthened when exposure occurred in the saccular phase. Studies suggest that the immature lung appears to be more susceptible to noxae in the saccular phase of development [71]. Furthermore, oxygen exposure to newborn rats during the saccular and early alveolar stages of lung development led to an immediate and persistent increase of oxidative stress [39]. Oxidative stress was associated with a persistent increase in the number of pulmonary immune cells. When monocytes migrate into the pulmonary tissue, they differentiate and become alveolar macrophages. Immunohistological staining for ED-1, a monocyte/macrophage marker, MPO for activated neutrophils, and gene expression analyses for chemoattractants for monocytes and neutrophils, respectively, showed early upregulation of MCP-1, MIP-2, and MIF, persistent until P15. The invading cells produce MCP-1, which acts as a chemoattractant and as an activator of monocytes [72], resulting in an enhanced monocyte recruitment. These cells also produce CINC-1, which is chemotactic for neutrophils, causing a secondary influx of neutrophils. This could explain the long-lasting presence of monocytes and neutrophils in the lung tissue after the recovery period. The fact that antineutrophilic [61] and antimacrophage [73] chemokine treatment prevented the influx of immune cells and reduced protein oxidation in newborn rats exposed to hyperoxia [39] significantly supports the essential role of the suppression of the hyperoxia-induced inflammatory response and abolishment of oxidative stress by caffeine.

The CD74 receptor mediates the binding of extracellular MIF [74]. The interaction of MIF, which is expressed by macrophages, other immune cells, and endothelial cells [75], with CD74 leads to the activation of several signaling transduction pathways involved in the regulating inflammation and immunity [76, 77]. In models of BPD, the interaction of MIF with CD74 expressed on the surface of alveolar macrophages resulted in MIF-induced neutrophil accumulation in the lung [78, 79]. High MIF mRNA and protein levels within the lungs of ARDS patients are associated with acute lung injury [80]. In human ARDS patients, treatment with recombinant MIF augmented proinflammatory cytokine secretion and an anti-MIF antibody treatment attenuated proinflammatory response, which suggests the role of MIF as a mediator of pulmonary inflammation [81]. Sun et al. [82] suggest a protective response in cases of sustained hyperoxia with a significant MIF increase in the murine model by reducing lung permeability, because for one thing the loss of MIF in the MIF knock out mice was associated with increased mortality and for another the MIF overexpression in transgenic mice with better survival. Oxidative stress led to high MIF expression and consequently to the induction of cytokine transcription, MIF being a key cell cycle regulator and influencing apoptosis. In contrast, low MIF expression resulted in reduced transcription of cytokines and apoptosis-relevant genes [79]. The regulatory functions of MIF and CD74 in pathogenesis of BPD [79] are still poorly understood.

Furthermore, our results revealed that exposure to hyperoxia directly resulted in elevated levels of proinflammatory cytokine mRNA, which could be ameliorated by caffeine delivery. After recovery, the expression of proinflammatory cytokines was comparable to the control group or revealed a significant reduction in transcription. The bimodal process of hyperoxia-induced lung injury results directly from oxygen toxicity [83] and from accumulation of inflammatory cells and mediators in the pulmonary tissue [84]. The decisive mediator function of cytokines became clear in transgenic cytokine mouse models for IL-1*β* [85] and IFN*γ* [86], which developed a BPD phenotype. High cytokine mRNA expressions are detected in hyperoxia-induced injury models [87, 88]. IL-10 is considered a major anti-inflammatory cytokine [89] with pleiotropic effects in the regulation of immune response and inflammation. IL-10 is known to reduce the secretion of proinflammatory cytokines, terminate inflammatory responses, and diminish lung injury [90]. In line with this, IL-10 mRNA was decreased directly after hyperoxia, but increased after recovery. Caffeine treatment showed an arresting effect only after 5 days of oxygen, but under room air, an impressive induction of mRNA expression was found at P15. Interestingly, BPD infants demonstrated a decrease of IL-10 in tracheal aspirates [91], and hyperoxia-exposed mice showed unchanged IL-10 mRNA levels [88], whereas exogenous IL-10 treatment alleviates hyperoxia-induced acute lung injury in mice, possibly by the diminished neutrophil recruitment and subsequent generation of cytokines [92]. Furthermore, IL-10 is produced by macrophages and regulatory T cells (Treg) and can inhibit the release of proinflammatory cytokines by innate immune cells. Treg may be also inducible after stimulating T cells in a specific cytokine milieu [93]. They can suppress or downregulate immune responses through suppression of autoreactive T cell responses or secretion of anti-inflammatory cytokines such as IL-10 [94].

It is acknowledged that NF*κ*B regulates the transcription of several genes related to the immune response to exogenous and endogenous noxae and thus plays an important role in inflammatory processes, cell survival, and developmental functions in the newborn lung [53]. Further, NF*κ*B transcription can be activated by hyperoxia followed by amplification of inflammatory response [84], and during the saccular stage, overexpressing NF*κ*B in the airway epithelium induces inflammation and disrupts lung development [95]. Oxidative stress rapidly activated the cellular pool of GCLC, which is important for the rate-limiting step in the formation of the antioxidant glutathione [96]. GCLC is transcriptionally controlled by Nrf2 and mediated by the NF*κ*B pathway, as important regulators of ROS defense [97]. These facts suggest that redox-sensitive transcription factors, such as NF*κ*B and Nrf2, are key mediators in oxidative stress-mediated pulmonary damage. This is consistent with our results that oxidative stress induced NF*κ*B and Nrf2 [39], coinciding with GCLC activation and with the proinflammatory responses. Caffeine had an inhibitory effect on the transcription of NF*κ*B subtypes and as well on GCLC, and thus, inflammatory cytokines and chemokines were decreased, underlining the anti-inflammatory effect of caffeine.

Caffeine reduced cell death, inflammation, and oxidative stress in the developing organism in hyperoxia-induced injury models [39, 45–47]. In line with other articles, additionally to the protective effects, undesired side effects, such as induction of chemokines and cytokines as well as modulation of the redox-sensitive transcription factor NF*κ*B, were also observed [45, 98]. As shown in this study, caffeine affected the immune response in the sense of a significant monocyte influx without additional oxidative stress. Dayanim et al. [99] observed increased cell death due to caffeine, which we could not confirm. A potential explanation of our results might be related to dose-dependent proinflammatory and anti-inflammatory effects. Valdez et al. reported that caffeine plasma levels outside the therapeutic range of 10 to 20 *μ*g/ml are associated with an increase of proinflammatory cytokines [100]. We measured at P3 and P5 plasma caffeine level between 8.8 and 11.2 *μ*g/ml, cited to the previous study [39], which is inside the therapeutically range [101]. However, it can be assumed that the plasma concentration was higher directly after application to P2 and P4, respectively. Hallmarks of chronic lung diseases vary; common features among oxidative stress are massive recruitment of inflammatory effector cells and the increased release of inflammatory mediators enhancing pulmonary injury. Inflammatory changes in the immature lung resulted in the disruption of pulmonary development and remodeling of the extracellular matrix [102]. Caffeine seems to be protectively preventing oxygen damage but also induced and modulated the immune response.

Caffeine is a nonspecific adenosine receptor antagonist. Adenosine *per se* is a signalling molecule increasingly produced upon cellular stress and an important key mediator through regulation of pulmonary inflammatory response and repair as well as alveolar development [54, 103]. Both caffeine and adenosine act through adenosine receptor subtypes (Adora1, 2a, 2b, and 3) [104], with main evidence in the lung for Adora1, 2a, and 2b. Under hyperoxic exposition, extracellular adenosine rapidly increases [105]. Elevated adenosine during acute lung injury minimized cytokine expression, immune cell infiltration, and vascular permeability, but persistent adenosine signalling in chronic lung injury increased cytokine secretion and immune cell influx in pulmonary tissue [106] as well as dysregulated tissue remodeling [54]. Caffeine blocks A1 and A2a adenosine receptors for natural ligand adenosine and may have caused partly the opposite effects of persistent adenosine [107]. An indication for the relevance of high adenosine concentrations in pathogenesis of lung injuries is the response enzyme to degraded adenosine is adenosine deaminase (ADA). A high adenosine level is associated with a high ADA serum level of preterm infants, which correlated with severity of disease [108]. Additionally, Adora2b is the most insensitive adenosine receptor, is expressed in low abundance, and is rarely achieved under physiological adenosine concentrations [109]. With higher adenosine sensitivity, Adora2a plays a crucial role during modulation of inflammatory response [110] and Adora1 revealed pro- and anti-inflammatory properties [111]. Our results showed a strong influence of both caffeine and oxygen on adenosine receptor transcription. While all three receptors are downregulated directly after hyperoxia, Adora2b showed an increased expression at P5. This could be interpreted as a cellular reaction to the increased adenosine secretion. Caffeine itself enhances the expression at P5 for all adenosine receptor subtypes. Caffeine with hyperoxia had little effect on adenosine receptor transcription after hyperoxia, except Adora1 at P5. Both hyperoxia and caffeine influence the transcription of the adenosine receptors. The Adora2a receptor subtype takes part in a negative feedback mechanism that limits both tissue-specific and systemic inflammatory responses [112]. Chemokines as being specifically regulated by Adora2a can modify the profile of inflammatory mediators of mononuclear cells and may limit the peak of an inflammatory response. Caffeine antagonism of Adora2a seems to modulate the production of inflammatory factor by neutrophils and reduces the inflammatory response to the oxidative stress.

Exposure to high oxygen leads to an inflammatory state within the lung, which is mediated by oxidative stress. The inflammatory response may be a result of a positive feedback cycle occurring in the production of ROS by inflammatory cells, which in turn recruits more inflammatory cells to the lung [69].

Possible working mechanisms for the effects of caffeine on hyperoxia-induced lung injury could be explained via the antioxidative caffeine metabolite 1,7 dimethyl-xanthine [113] and via the anti-inflammatory decrease of neutrophil chemokine level mediated to the antagonism of the Adora2a receptor [114]. In combination of these facts, caffeine is a potent free radical scavenger and adenosine receptor antagonist, and so caffeine reduced pulmonary tissue damage by modulation of cell death-associated mediators and of immune response. The antioxidative effect, the corresponding reduced oxidative stress response [39], and a direct effect of caffeine via the adenosine receptor subtypes probably modulated inflammation-associated pathways and contributed to the protective effect of caffeine in this BPD model. However, an in-depth mechanistic understanding of the crosstalk between the intracellular stress signalling pathways and inflammatory responses and any shift in the balance of inflammatory stress responses or reduction of oxidative stress may lead to a better outcome in terms of respiratory disease progression of preterm infants.

## 5. Conclusions

The level of knowledge is that oxidative stress resulted from supportive oxygen therapy in premature infants, followed by consequences such as inflammation and impairment on lung structure and function. Inflammation and oxidative stress are important contributors to the pathogenesis of BPD. No targeted therapy currently exist to prevent pulmonary inflammation and injury due oxidative stress. Caffeine is the widespread drug of choice to prevent and treat apnoea of prematurity. Caffeine therapy shortens the duration of mechanical ventilation in very and extremely premature infants and reduces the incidence of BPD. In present, clinically relevant questions are aimed at timing and duration of caffeine therapy, the targeted caffeine plasma concentration, and the consideration of prophylactic use of caffeine, which is focused on respiratory outcome in the adolescent and adulthood. To date, there is a consequent uncertainty of the long-term benefit or risk ratio of caffeine therapy.

Our study provides further evidence that caffeine exerts protective actions against hyperoxia-induced pulmonary injury, independent of oxygen exposure duration and with regard to the stages of lung development. Caffeine acts on anti-inflammatory, anti-apoptotic, and altered inflammatory responses and adenosine receptor expression. Furthermore, high oxygen concentrations also caused a sustained cell death rate beyond the recovery phase in ambient air in connection with increased chemokine levels. This might indicate an oxygen-induced long-term modulation of the immune response and thus be causal for persistent lung damage in premature infants. However, caffeine without oxidative stress unexpectedly demonstrated an overwhelming inflammatory response when applied during the saccular phase of lung development. This seems to be an impressive counterargument to preventive caffeine supplementation in extreme and very premature neonates in the absence of oxidative stress. Future research should aim at immunomodulatory effects of oxygen per se but also of caffeine, for a better understanding in the development of new therapeutic strategies. Our findings proved the relationship between oxidative stress induced by hyperoxia and subsequent inflammatory response and helped to understand the higher risk of respiratory impairments in the later life of preterm infants.

## Figures and Tables

**Table 1:** Sequences of oligonucleotides.

	Oligonucleotide sequence 5′-3′	Accession no.
*Adora1*		
Forward	GGATCGATACCTCCGAGTCAAG	NM_017155.2
Reverse	AATCCAGCAGCCAGCTATGG	
Probe	TCTCCGGTACAAGACAGT	
*Adora2a*		
Forward	GGGAGCCAGAGCAAGAGGTA	NM_053294.3
Reverse	CTGCATCTCCCAAAATCATGAC	
Probe	AGAACCCTGAAGCCGAGA	
*Adora2b*		
Forward	TCGTGCTGGTGCTCACACA	NM_017161.1
Reverse	TCGTGTTCCAGTGACCAAACC	
Probe	ATCTTTAGCCTCTTGGCGGT	
*AIF*		
Forward	CACAAAGACACTGCAGTTCAGACA	NM_031356.1
Reverse	AGGTCCTGAGCAGAGACATAGAAAG	
Probe	AGAAGCATCTATTTCCAGCC	
*Casp3*		
Forward	ACAGTGGAACTGACGATGATATGG	NM_012922.2
Reverse	AATAGTAACCGGGTGCGGTAGA	
Probe	ATGCCAGAAGATACCAGTGG	
*CD74*		
Forward	CCCGTGAAGAATGTTACCAA	NM_013069.2
Reverse	AACAGCCACTGTTTCATCCA	
Probe	ATGAATGGTCTGGACTGGAA	
*CINC-1*		
Forward	GCTGTCAGTGCCTGCAGACA	NM_030845.1
Reverse	GACCATTCTTGAGTGTGGCTATGA	
Probe	CACTTCAAGAACATCCAGAG	
*GCLC*		
Forward	GGAGGACAACATGAGGAAACG	NM_012815.2
Reverse	GCTCTGGCAGTGTGAATCCA	
Probe	TCAGGCTCTTTGCACGATAA	
*HPRT*		
Forward	GGAAAGAACGTCTTGATTGTTGAA	NM_012583.2
Reverse	CCAACACTTCGAGAGGTCCTTTT	
Probe	CTTTCCTTGGTCAAGCAGTACAGCCCC	
*IFNγ*		
Forward	CGGACCAGAGACCCTTTGC	NM_001082479.1
Reverse	GCCTGTGGGCTTGTTGAAGT	
Probe	CTCTTCAGTTCGTGTGTGG	
*IL-1α*		
Forward	GAAGATGACCTGGAGGCCATAG	NM_017019.1
Reverse	TCCTGCTTGACGATCCTTATCA	
Probe	AAGAGACCATCCAACCCAGA	
*IL-1β*		
Forward	CTCCACCTCAATGGACAGAACA	NM_031512.2
Reverse	CACAGGGATTTTGTCGTTGCT	
Probe	CTCCATGAGCTTTGTACAAG	
*IL-10*		
Forward	CCCTGGGAGAGAAGCTGAAGA	NM_012854.2
Reverse	GCTCCACTGCCTTGCTTTTATT	
Probe	CATCGATTTCTCCCCTGTGA	
*MCP-1*		
Forward	AGCATCCACGTGCTGTCTCA	NM_031530.1
Reverse	GCCGACTCATTGGGATCATC	
Probe	AGATGCAGTTAATGCCCCAC	
*MIF*		
Forward	GCAAGCCGGCACAGTACAT	NM_031051.1
Reverse	GCTCGTGCCACTAAAAGTCATG	
Probe	CAGTGCACGTGGTCC	
*MIP-2*		
Forward	CCTACCAAGGGTTGACTTCAAGA	NM_053647.1
Reverse	GCTTCAGGGTTGAGACAAACTTC	
Probe	AGACAGAAGTCATAGCCACT	
*NFκB1*		
Forward	GACCCAAGGACATGGTGGTT	NM_001276711.1
Reverse	TCATCCGTGCTTCCAGTGTTT	
Probe	CTGGGAATACTTCACGTGAC	
*NFκB2*		
Forward	GCCTAAACAGCGAGGCTTCA	NM_001008349.1
Reverse	TCTTCCGGCCCTTCTCACT	
Probe	TTTCGATATGGCTGTGAAGG	
*TNFα*		
Forward	CCCCCAATCTGTGTCCTTCTAAC	NM_012675.3
Reverse	CGTCTCGTGTGTTTCTGAGCAT	
Probe	TAGAAAGGGAATTGTGGCTC	

## Data Availability

The data used to support the findings of this study are available from the corresponding author upon request.
